# BLOBFISH: bipartite limited subnetworks from multiple observations using breadth-first search with constrained hops

**DOI:** 10.1093/bioinformatics/btag677

**Published:** 2026-09-10

**Authors:** Tara Eicher, Marouen Ben Guebila, John Quackenbush

**Affiliations:** Department of Biostatistics, T.H. Chan School of Public Health, Harvard University, Boston, MA 02115, United States; Department of Biostatistics, T.H. Chan School of Public Health, Harvard University, Boston, MA 02115, United States; Department of Biostatistics, T.H. Chan School of Public Health, Harvard University, Boston, MA 02115, United States; Channing Division of Network Medicine, Brigham and Women’s Hospital, Boston, MA 02115, United States

## Abstract

**Motivation:**

In analyzing biological network models, such as gene regulatory networks, a common question is how members of a particular set of genes are connected. For example, one might want to explore network relationships between a set of differentially expressed genes, a gene set previously reported in the literature, or elements of one or more pathways. BLOBFISH uses a breadth-first search algorithm adapted to bipartite graphs to identify a compact subnetwork connecting the members of a pre-specified set of genes, providing a regulatory context that can shed light on specific mechanisms involved in a phenotype and its development.

**Results:**

We demonstrate the use of BLOBFISH to extract connected subnetworks between candidate nodes in and gene regulatory and eQTL networks reflecting tissue specificity using publicly available data from the Genotype Tissue Expression (GTEx) project.

**Availability:**

Source code is available from GR as part of the netZooR R package (v1.6) (https://github.com/netZoo/netZooR). Replication scripts are available from https://github.com/QuackenbushLab/BLOBFISH_paper_scripts. eQTL networks are available from Zenodo (doi: 10.5281/zenodo.20820178). LIONESS networks are available from GRAND (https://grand.networkmedicine.org/tissues/).

## 1 Introduction

It is common to think of biological states as being defined by specific genetic variants or patterns of expression, but we have come to recognize that the real drivers of phenotypes are complex networks of interacting cellular elements that drive the processes specific to each phenotype and that characterize the transitions between states. There are many tools for inferring networks that represent specific aspects of biological systems and these have proven their value in characterizing the relationships between genes and other entities in the context of understanding phenotypic differences (Eicher *et al*. 2020, Weighill *et al*. 2021). Although unipartite networks such as gene co-expression based on correlations or partial correlations are frequently used (Langfelder and Horvath 2008), bipartite networks provide the opportunity to identify interactions between different types of cellular elements, including genes and their regulators. Examples of bipartite networks include expression quantitative trait loci (eQTL) networks (Platig *et al*. 2016, Fagny *et al*. 2017), gene regulatory networks (GRNs) (Glass *et al*. 2013, Kuijjer *et al*. 2020), and multi-omic partial correlation networks (Eicher *et al*. 2023).

Although networks can capture genome-scale interactions, interpreting such results can be challenging, particularly in the context of exploring a hypothesis regarding a subset of genes thought to be relevant to a phenotype under study (Edge *et al*. 2018). Interest in examining network relationships between a subset of “interesting” genes has motivated the development of tools to evaluate connectivity between a set of “seed” nodes, including methods based on random walks with restarts (Liao *et al*. 2020, Chakrabarty *et al*. 2021) and Steiner trees (White and Ma’ayan 2007). However, most subgraph extracting methods have a stochastic component so that multiple applications to the same starting network, or changes in how the network is recorded, may lead to different results. There are also deterministic methods that can identify unique paths connecting seed nodes, including some based on shortest paths (Li *et al*. 2012, Keane *et al*. 2015), but in applications such as drug targeting and pathway analysis, where biological systems often have redundancies, deterministic methods can miss multiple connecting paths that define more biologically relevant patterns of interactivity (Ogris *et al*. 2022, Lee *et al*. 2023). While one software tool, Genes2Networks, allows for redundancy of paths, it is not actively maintained (Berger *et al*. 2007).

A key hypothesis in network analysis is that networks not only differ between phenotypes but also vary among individuals within any specific phenotypic group. Methods such LIONESS (Kuijjer *et al*. 2019), BONOBO (Saha *et al*. 2023), and SWEET (Chen *et al*. 2023), used in conjunction with other methods, can infer networks for each individual in a population and there are a number of methods that can be used to for single-cell specific network inference (such as those reviewed by Pratapa *et al*. (2020)). This suggests that a useful strategy for mapping subnetworks connecting genes would be to identify robust edges that are consistent across individuals, thereby providing greater confidence in the identified connectivity between genes. None of the aforementioned methods for evaluating connectivity between seed nodes is designed to identify robust edges between multiple networks, making this an important avenue for method development.

## 2 Methods

We developed BLOBFISH (*B*ipartite *L*imited Subnetworks from Multiple *O*bservations using *B*readth-*Fi*rst *S*earch with Constrained *H*ops) to find a robust subnetwork connecting a seed set of nodes in collections of bipartite networks generated from individual samples to find patterns of connection consistent across observations. Conceptually, BLOBFISH is based on the idea that each node in a bipartite network has a sphere of influence that can be obtained using breadth-first search with a user-defined constraint on the number of allowed hops between nodes such that the connectivity between two nodes represents paths to shared nodes within each node’s sphere of influence. This allows multiple paths to connect individual nodes in a way that mirrors the redundancy and shared functionality of genes known to exist in biological networks. To provide confidence in the resulting subnetworks, BLOBFISH requires that all edges in the subnetwork must have statistically significant weights when compared against a null model. Users provide an appropriate null model representing a distribution of insignificant edge weights as well as a *P*-value cutoff *α* for determining significance, such as α=0.05—smaller values of *α* are expected to yield more sparse networks. Because BLOBFISH operates on bipartite graphs, all connecting paths can be found by evaluating only shared nodes equidistant from a pair of seed nodes, as stated formally in Theorem 1 (see Supplementary File 1 for proof, available as supplementary data at *Bioinformatics* online).

Theorem 1. Any path $P_{b,c}$ between nodes *b* and *c* in a network *G* contains a node *a* such that $D_{a,b}=D_{a,c}$, where $D_{i,j}$ represents the shortest unweighted graph distance between two nodes *i* and *j*.

By making use of this property in addition to being deterministic, BLOBFISH provides runtime advantages over a naïve exploration of all possible paths across spheres of influence, with an empirical bound of $O(h{\left|V\right|}^{3})$ for whole-genome and whole-transcriptome data sets, where *h* is the number of hops included in the search and *V* is the set of all nodes in the network. The BLOBFISH algorithm is described as pseudocode in Supplementary File 1, available as supplementary data at *Bioinformatics* online together with analysis of some of its performance characteristics, including in-depth runtime analysis.

BLOBFISH takes as input a collection of gene regulatory networks or other bipartite biological networks derived by applying a specific network inference method to an experimental dataset and a null network distribution by using multiple randomizations of the expression levels (or other biological measurements) and using the same network inference method to generate a collection of network models. BLOBFISH analyzes the collection of individual-sample network graphs and, for a set of seed genes, computes significance using a Wilcoxon rank-sum test (for a single sample, the empirical *P*-value is used instead). BLOBFISH uses a breadth-first search (BFS) to identify neighborhoods for each gene in the reduced network, limiting its search based on hop constraint *h*. BFS explores gene nodes and regulatory edges connecting them systematically starting from each of the seed genes. It explores all neighbors of the current node before moving to the next level of neighbors, ensuring that all nodes are visited in order of their distance from the source, resulting in a sphere of influence for each gene based on a user-determined parameter. The algorithm then finds all possible paths connecting the genes based on the overlap of the collective spheres of influence. For instance, if the user selects h=2 for a GRN, BLOBFISH will return a network including only TFs co-regulating multiple genes in the seed set, whereas h=4 will also include both genes that are co-regulated with multiple genes in the seed set and the TFs that co-regulate them. For gene regulatory networks, h=2 identifies transcription factors directly co-regulating multiple seed genes. Increasing the search to h=4 permits identification of genes connected through two regulatory layers, producing larger subnetworks that capture broader regulatory neighborhoods at the expense of specificity. The proper balance in each study depends on the user’s overall goal. See Fig. S1, available as supplementary data at *Bioinformatics* online for a schematic of BLOBFISH.

BLOBFISH is intended for analysis of bipartite biological networks when the goal is to identify mechanistic connections among a predefined set of biologically relevant seed nodes. Typical applications include connecting differentially expressed genes within gene regulatory networks, identifying regulatory subnetworks underlying pathway members, or linking phenotype-associated genes through shared regulators in GRNs or SNP variants in eQTL networks. The method is most useful after genome-wide network inference has identified candidate genes, providing an interpretable regulatory context rather than performing network inference itself.

BLOBFISH is integrated into the *netZooR* R package (v1.6) (https://github.com/netZoo/netZooR) (Ben Guebila *et al*. 2023) and can be run using a call to the function *RunBLOBFISH*(). The additional functions *PlotNetwork*() and *GenerateNullPANDADistribution*() support plotting the subnetwork and generating the null distribution for bipartite networks generated using PANDA’s message-passing framework or those of its related methods (Glass *et al*. 2013, Kuijjer *et al*. 2020, Sonawane *et al*. 2021, Weighill *et al*. 2022, Osorio *et al*. 2024); the latter function could be easily modified to use other network inference methods. BLOBFISH depends on the R packages *igraph* and *matrixTests*. The script used in our analysis is available from https://github.com/QuackenbushLab/BLOBFISH_paper_scripts.

## 3 Results

As a test of BLOBFISH, we downloaded from the GRAND database (Ben Guebila *et al*. 2022) GRN models that had been inferred by applying PANDA+LIONESS to GTEx data (Lonsdale *et al*. 2013, Lopes-Ramos *et al*. 2020) for five tissues (subcutaneous adipose, skeletal muscle, lung tissue, skin, and aorta). To minimize influence of age and biological sex on the GRNs, we used only networks generated for males between the ages of 20–29, resulting in *n *= 18 for subcutaneous adipose, *n *= 27 for skeletal muscle, *n *= 15 for lung tissue, *n *= 31 for skin, and *n *= 15 for aorta. We used 26 genes reported to be typical of skeletal muscle by Bortoluzzi *et al*. (2000) and seven genes involved in adipogenesis (Ou-Yang and Dai 2023) as seed gene sets (described in Supplementary Tables 1 and 2, available as supplementary data at *Bioinformatics* online).

We ran BLOBFISH using both the skeletal muscle and adipogenesis seed gene sets on each tissue-specific collection of bipartite gene regulatory networks using a null distribution generated using *GenerateNullPANDADistribution*(), *α*  =  0.05, and *h *= 2; full subnetworks can be found in Supplementary Tables 3–7, available as supplementary data at *Bioinformatics* online. To evaluate only the tissue-specific connectivity between genes in the seed set and exclude connectivity shared between tissues, we retained only edges that were exclusive to each tissue type’s subnetwork. The results can be found in Supplementary Tables 8–12, available as supplementary data at *Bioinformatics* online.

To evaluate BLOBFISH beyond the PANDA+LIONESS context, we applied BLOBFISH to eQTLs inferred from the same GTEx tissues (subcutaneous adipose, skeletal muscle, lung, skin, and aorta) using all available samples and the same set of genes. Generation and processing of eQTLs is described in detail by Stone *et al*. (2025). Briefly, *cis*-eQTLs and *trans*-eQTLs were inferred using the *MatrixEQTL* R package for each GTEx (v 8.0) tissue. eQTLs with *FDR*-adjusted *P*-value < 0.25 were retained. Because the networks were pre-filtered, we did not apply additional significance filtering when running BLOBFISH. We then mapped the SNPs to genes using GENCODE (v 49, basic gene annotation, chromosomal regions only). While GRN analyses were restricted to young males to reduce biological heterogeneity, we used independently generated networks for eQTL analysis, which allowed us to demonstrate the effects of sex and age. Despite these differing cohorts, BLOBFISH recovered biologically consistent tissue-specific subnetworks in both analyses, demonstrating the power of the approach. Tissue-specific connectivity can be found in Supplementary Tables 13–17 and Fig. S2, available as supplementary data at *Bioinformatics* online.

### 3.1 Methodological validation

To assess the significance of the connectivity we found among gene sets associated with skeletal muscle development and adipogenesis in the relevant tissues, we used BLOBFISH to assess these and 100 random sets of 33 (26 + 7) genes in all five tissues for which we had GRN subnetwork models.

**Figure 1:**
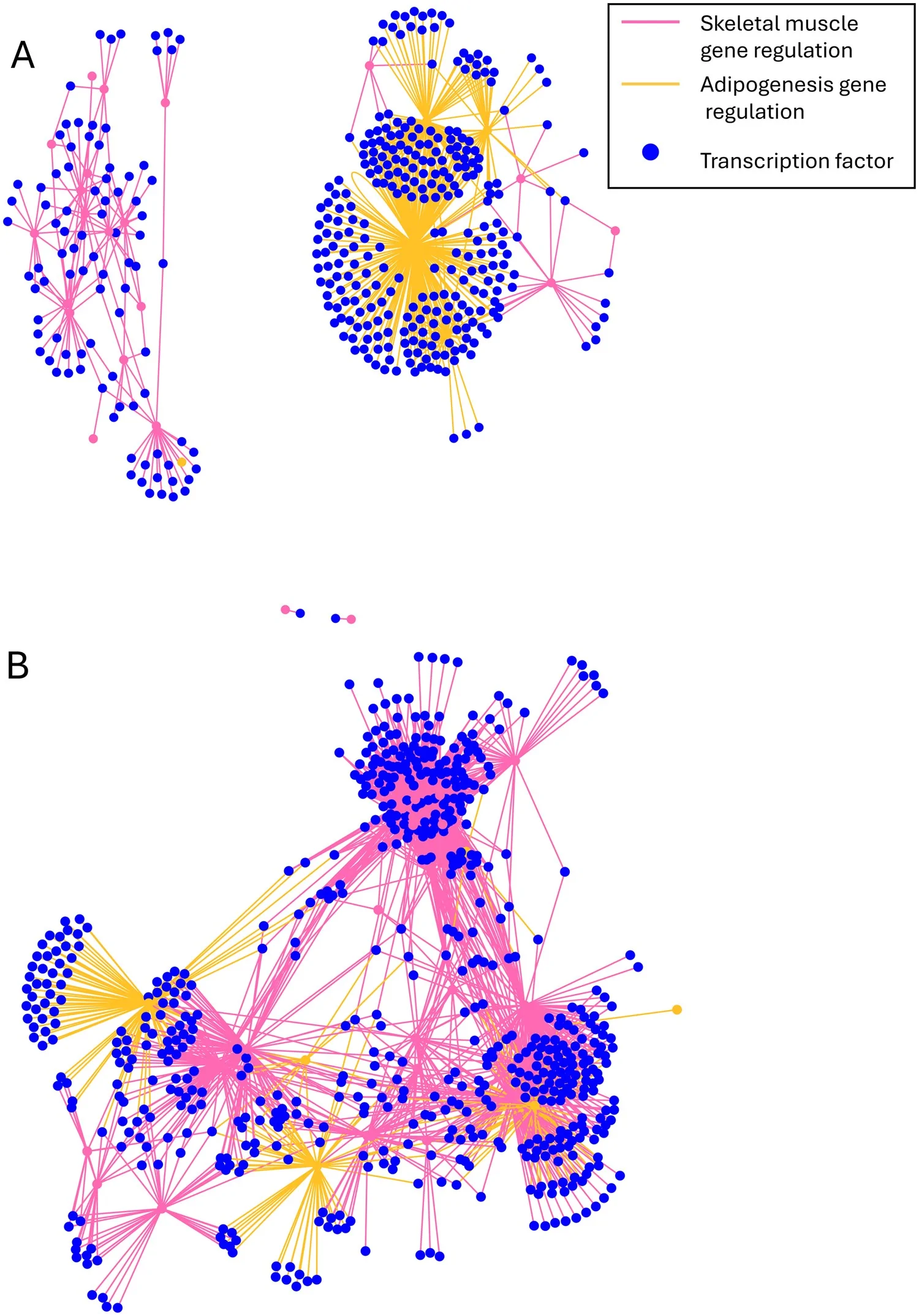
Tissue-specific gene regulatory subnetworks for GTEx skeletal muscle samples for males aged 20–29 years. Panels represent (A) subcutaneous adipose and (B) skeletal muscle, revealing more co-regulatory connections between skeletal-muscle-associated genes (pink) in skeletal muscle and more co-regulatory connections between adipogenesis-associated genes (gold) in subcutaneous adipose.

We expect that few if any genes are expressed in only a single tissue and many genes are expressed in a large number of tissues; indeed, for each set of seed genes, we found subnetworks linking some subset of those seed genes in each of the five tissues in nearly every iteration. But we also expect phenotype-specific sets of genes to be highly connected in the relevant tissue since some level of coordinated regulation of these genes defines, or is defined by, that tissue. Consistent with this, the mean count of TFs co-regulating each pair of skeletal-muscle-associated genes was highest in the skeletal-muscle-specific subnetwork and the mean count of TFs co-regulating each pair of adipogenesis-associated genes was highest in the subcutaneous-adipose-specific subnetwork (Fig. 1 and Table 1; see Fig. S3, available as supplementary data at *Bioinformatics* online for the other tissue-specific subnetworks).

**Table 1:** ** **The mean number of TFs co-regulating skeletal-muscle-associated and adipogenesis-associated genes in each tissue, compared to the mean number of TFs co-regulating random subsets of genes.

	Sub. adipose	Skel. muscle	Lung tissue	Skin	Aorta
µ_r_^a^, *σ^b^*	0.48, *0.35*	6.96, *2.18*	0.42, *0.24*	1.14, *0.59*	0.25, *0.20*
µ_s_, *z*^c^	0.35, *−0.37*	**12.50, *2.54***	0.51, *0.38*	0.26, *−1.49*	0.10, *−0.75*
µ_a_, *z*		0.48, *−2.97*	0.00, *−1.75*	1.00, *−0.24*	0.43, *0.90*

a Mean shared transcription factor counts for random (*r*), skeletal-muscle-associated (*s*), and adipogenesis-associated (*a*) gene sets, respectively.

b Standard deviation of shared transcription factor counts across random gene sets.

c *z*-Score when computed against µ_rand_ and σ.

Bolded values represent results for adipogenesis-associated genes in subcutaneous adipose and skeletal-muscle-associated genes in skeletal muscle, respectively.

Further, in the relevant tissue, the mean counts for tissue-specific gene sets were considerably higher (with statistically significant *z*-scores) than the mean counts obtained from the random gene sets. Mean counts between genes associated with skeletal muscle were obtained by computing, for each pair of genes, the number of TFs regulating both genes and then averaging over all pairs, and likewise for genes associated with adipogenesis. In contrast, the TF connectivity for the same adipose and muscle gene sets was essentially at background levels in lung, skin, and aorta based on its distribution in random gene sets. This suggests that BLOBFISH can extract meaningful regulatory subnetwork connections between a tissue- or disease-specific set of genes when applied to GRNs inferred in a relevant tissue using PANDA+LIONESS.

We further compared BLOBFISH to an in-house implementation of the shortest paths (SP) algorithm described by Keane *et al.* (which is not available as a software package) (Keane *et al*. 2015). Because SP does not perform significance filtering, we first filtered the networks using BLOBFISH to retain only significant, binarized edges, then input these into the algorithm (results shown in Fig. S4, Supplementary Tables 18–22, available as supplementary data at *Bioinformatics* online). The SP algorithm found more TFs shared between adipogenesis genes in lung tissue and in skeletal muscle than in subcutaneous adipose, illustrating that characterization of context-specific biological network features requires inclusion of redundant paths (Table S23, available as supplementary data at *Bioinformatics* online).

Finally, we applied BLOBFISH to the eQTL networks obtained from subcutaneous adipose, skeletal muscle, and lung tissues for *h *= 2, *h *= 4, and *h *= 6 to evaluate the impact of hop count on network scale and performance. While increases in runtime were manageable from *h *= 2 (average 5.60 minutes) to *h *= 4 (average 11.60 minutes), average runtime at *h *= 6 increased to 29.67 hours and resulted in networks with over 1,000 nodes, illustrating that *h *> 4 may introduce scalability concerns for whole-ome networks (Table S24, available as supplementary data at *Bioinformatics* online).

### 3.2 Biological interpretation

Beyond the tissue specificity we found for connected subnetworks of tissue-relevant gene sets, one would expect that the additional genes connected to the seed set found by BLOBFISH might further help explain the phenotype that the input gene set was meant to capture. To test this, we took the BLOBFISH subnetworks for each tissue generated using the combined skeletal muscle and adipogenesis genes as seeds, and for each, performed pre-ranked gene set enrichment analysis on the TFs and genes using the *fgsea* R package (Korotkevich *et al*. 2021) with *FDR*-adjusted *P*-value < 0.05 and gene sets from the Molecular signatures Database (MSigDB, version 2023.2) (Subramanian *et al*. 2005, Liberzon *et al*. 2015); the number of edges adjacent to each TF or gene was used as the input ranking score. We then compared these results to pathways enriched in each tissue using the log_2_(fold change) of the expression levels of the seed as input.

The only functional class that was enriched in the subcutaneous adipose tissue subnetwork was adipogenesis, but this was not surprising given the small number of adipogenesis-relevant genes in the input (seven). In comparison, pathway analysis using expression of the seed resulted in enrichment of PPAR signaling, one step in adipogenesis (Ghaben and Scherer 2019). In the skeletal muscle subnetwork, we found enrichment for both muscle contraction and Parkinson’s Disease pathways, whereas no pathways were enriched using gene expression data for this seed set. Impaired skeletal muscle function is a hallmark of Parkinson’s Disease (Murphy and Lynch 2023). This result provides further evidence that BLOBFISH can use tissue-specific gene regulatory networks to aggregate sets of genes linked through common regulators that share a common biological function. The results for all tissues are presented in Supplementary Tables 25–29, available as supplementary data at *Bioinformatics* online (for network-based analysis) and 30–34 (for expression-based analysis).

We further examined hub TFs in the context of their established regulatory associations, considering TFs with at least ten skeletal-muscle-specific or adipose-specific genes as hubs. These included *ARID2*, *ARNT2*, *ASCL1*, *BCL6B*, *CEBPA*, and *ELK3* in skeletal muscle and *ATF4* in subcutaneous adipose. We found that *ARID2* is associated with the growth and development of muscle fibers, *BCL6B* is exercise-responsive, *CEBPA* regulates skeletal muscle atrophy, and *ATF4* orchestrates obesity-associated metabolic inflammation in adipose tissue macrophages (Wu *et al*. 2023, Lan *et al*. 2024, Luo *et al*. 2024, Bruss *et al*. 2025). In addition to finding enrichment of expected functional classes, BLOBFISH also identified tissue-specific TF activity that has been reported in the literature.

eQTL-based BLOBFISH analysis identified SNPs that target only skeletal muscle genes in all tissues; further analysis revealed that this was due to adipogenesis genes specific to adipose tissue being targeted by independent SNPs (Fig. S5, available as supplementary data at *Bioinformatics* online). However, the subnetworks revealed biologically meaningful tissue-specific effects of SNPs on these genes and were consistent with the PANDA-LIONESS networks. In skeletal muscle, desmin (*DES*) was co-targeted with α-tubulin by a SNP on *SPEG*, consistent with the known roles *DES* and *SPEG* in focal adhesion in skeletal muscle and in stabilizing microtubules, which are formed from α-tubulin (Luo *et al*. 2021, Salomon *et al*. 2022). In contrast, adipose tissue was characterized by co-regulation of mitochondrial-function-associated gene *MB* and vasculature-associated gene *TNNC1* by *OAT*, consistent with the role of *OAT* in both mitochondrial metabolism and vascular smooth muscle cell growth (Durante *et al*. 2001, Li and Hwang 2015, Ginguay *et al*. 2017, Christen *et al*. 2022). The PANDA-LIONESS and eQTL networks were consistent in that α-tubulin genes were heavily targeted across multiple tissues, reflecting their known role in multiple cellular functions (Binarova and Tuszynski 2019).

## 4 Discussion

A frequent question in the analysis of any biologically interesting phenotype is how subsets of genes previously identified as relevant interact with each other within the context of the regulatory processes that ultimately define each biological state. Methods that attempt to connect genes within those subsets, including methods using correlation analysis, fail to provide information on the broader regulatory context in which these genes exist. Genome-wide network inference methods that provide insight into regulatory processes, such as GRN inference and eQTL analyses, can offer deeper understanding of the factors influencing expression, but interpreting the complex connections across many cellular elements within those networks can be challenging.

BLOBFISH simplifies the problem of subnetwork identification and interpretation by using regulatory network bipartite graphs to find a minimal set of elements (such as genes and TFs or genes and SNPs) that provide plausible mechanistic and biological links between a candidate gene set. The support in BLOBFISH for bespoke networks and null distributions allows users to identify subnetworks in bipartite graphs of varying densities, as demonstrated with our application to relatively dense PANDA-LIONESS networks and the much sparser eQTL networks.

Many other network analysis methods use degree-based analyses, differential targeting analysis, or statistical comparisons of network edge weights to identify elements that differ between states—often yielding a set of candidate “differential” nodes. By providing a way to identify connections among biologically “interesting” genes, BLOBFISH complements these methods and fills an important methodological gap by extracting meaningful subnetworks that balance the comprehensiveness of genome-wide networks with the interpretability of a focused set of regulatory processes.

## Supplementary Material

btag677_Supplementary_Data

## Data Availability

Source code is available from GR as part of the netZooR R package (v1.6) (https://github.com/netZoo/netZooR). Replication scripts are available from https://github.com/QuackenbushLab/BLOBFISH_paper_scripts. eQTL networks are available from Zenodo (doi: 10.5281/zenodo.20820178). LIONESS networks are available from GRAND (https://grand.networkmedicine.org/tissues/).
