# Supplementary material for: BLOBFISH: bipartite limited subnetworks from multiple observations using breadth-first search with constrained hops
Source: Bioinformatics. 2026 Sep 10;42(9):btag677. doi: 10.1093/bioinformatics/btag677 (PMC13612173; doi:10.1093/bioinformatics/btag677)
Supplement: Supplementary file 1 [file btag677_supplementary_data.zip › Supplementary_File_1_revision2_final_clean.docx]

# Null Model

We define the null network model as a model in which, by design, no biological signal is expected to be present. In the case of PANDA-like networks such as those used in our analysis, we constructed the null by retaining the known transcription factor binding motifs (motif) and protein-protein interactions (PPI) between transcription factors (which are constant across all samples) and generating ten random gene expression networks, where the expression is drawn from a normal distribution. In these random gene expression networks, the expression levels of any two genes are generated independently; therefore, they are expected, on average, to contain only uncorrelated or spuriously correlated genes. Each random gene expression network is then input into PANDA along with the motif and PPI. After running PANDA, the edges comprising the motif and PPI were removed from the final networks, and the null distribution of edge weights was represented as the distribution of edge weights remaining in the final networks.

# Pseudocode

The BLOBFISH algorithm is described as pseudocode below, where *n* is the number of observations, *S* is an aggregate weighted adjacency list with $\left| E \right|$ rows and 2 + *n* columns, where columns 1 and 2 represent source and target nodes and columns 3 through 2 + *n* represent weights, *G* is the seed set, *α* is the significance cutoff for statistical testing, ∅ is a set of edge weights obtained from the null model, and *h* is the maximum number of hops allowed to connect a pair of genes in *G*.

```
function BLOBFISH(S,G,α, Ø,h):
   SphereOfInfluence := # List of lists of edge sets, with
                        #dimensions |G| and h/2
   // Find all significant edges.
   SigEdges := []
   For edge in 1..|E|:
      If FDR(pval(Wilcox.test(S[e, 3:n+2], Ø)) < α:
         SigEdges += e

   // Obtain the sphere of influence for each starting gene.
   For g in G:
	Start := g
Explored := []
For i in 1..(h/2): # Ranging from 1 to h/2
   SphereOfInfluence[g][i]:= BFS(S,Start)
```

Explored := Explored ∪ Start

```
   Start := SphereOfInfluence – Explored

   // Find all paths connecting genes’ sphere of influence.
   Subnet := []
   For g in G:
	For γ in G – g:
	   For i in 1..(h/2):
```

Subnet += SigSubnet[g][i] ∩ SigSubnet[γ][i]

return Subnet

# Analysis of Runtime from Pseudocode

**Lines 1-4:** The initialization of SigEdges and SphereOfInfluence are constant-time operations $O\left( 1 \right)$. Constant-time operations do not affect the bound.

**Lines 5-7:** All statements in the loop For edge in 1..|E|:run in $O\left( n\log n \right)$, the time to sort the null and test distributions when computing a Wilcoxon rank-sum test. Therefore, the total running time of the loop is $O\left( n\log\left( n \right)|E| \right)$

**Lines 10-16:** Because the initialization of Start and Explored are constant time operations, the cost of lines 13-16 (For i in 1..(h/2):) is multiplied by $\left| G \right|$. All set operations are $O(\left| V \right|)$, making the runtime of lines 13-16 $O\left( h\left| V \right| \right)$ and the total runtime of lines 10-16 $O\left( h\left| G \right|\left| V \right| \right)$*.*

**Lines 19-24:** Because line 19 is a constant time operation, the cost of lines 21-23 (For γ in G – g:) is multiplied by $\left| G \right|$, which in turn is the cost of lines 22-23 (For i in 1..(h/2):) multiplied by $\left| G \right|$. Therefore, the runtime of lines 22-23 is $O\left( h\left| V \right| \right)$, the runtime of lines 21-23 is $O\left( h\left| G \right|\left| V \right| \right)$, and the runtime of lines 19-24 is $O\left( h\left| G \right|^{2}\left| V \right| \right)$*.*

Because $h\left| G \right|\left| V \right|=O\left( h\left| G \right|^{2}\left| V \right| \right),$ $|E|\leq{|V|}^{2}$, and $|G|\leq|V|$, the total runtime of the algorithm is $O\left( h\left| G \right|^{2}\left| V \right|+n\log\left( n \right)|E| \right)=O\left( h\left| G \right|^{2}\left| V \right|+n\log\left( n \right){|V|}^{2} \right)=O\left( h\left| V \right|^{3}+n\log\left( n \right){|V|}^{2} \right)$.

Empirically, $n\log\left( n \right)\ll|V|$ for whole-omics data sets in which $n$ represents the number of samples and $|V|$ the number of genes in the genome or transcripts in the transcriptome. Moreover, because we limit $h\leq|V|$, we can expect the total runtime to be $O\left( h\left| V \right|^{3} \right)$ in this scenario, which is polynomial in $\left| V \right|$.

# Theorem 1

Any path $P_{b,c}$ between nodes *b* and *c* in *G* contains a node *a* such that $D_{a,b}=D_{a,c}$, where $D_{i,j}$ represents the shortest unweighted graph distance between two nodes *i* and *j*.

**Proof:**

Because *b* and *c* are of the same node type in a bipartite network, $P_{b,c}$ must contain an odd number of intermediate nodes and an even number of connecting edges, i.e., $mod\left( D_{b,c},2 \right)=0$.

Further, we have that $D_{b,c}=D_{c,b}$.

Then, traversal along the first $\frac{D_{b,c}}{2}$ edges in $P_{b,c}$ starting at *b* results in a path $P_{b,x}$where $x\notin\left\{ a,b \right\}$.

Further, backwards traversal along the last $\frac{D_{b,c}}{2}$ edges in $P_{b,c}$ starting at *c* results in a path $P_{c,y}$where $y\notin\left\{ a,b \right\}$.

Therefore, $D_{b,x}=D_{c,y}=\frac{D_{b,c}}{2}$. If $x=y$, then *x* satisfies the condition for node *a*.

We can then assume for purposes of contradiction that $x\neq y$.

Then either *x* is not connected to *y* or $D_{x,y}>0$.

Because $P_{b,c}$ contains both *x* and *y*, not all edges in $P_{b,c}$ are connected, contradicting its definition as a path.

If $D_{x,y}>0$, then $D_{b,c}=D_{b,x}+D_{x,y}+D_{c,y}>D_{b,x}+D_{c,y}$.

Substitution yields $D_{b,c}>\frac{D_{b,c}}{2}+\frac{D_{b,c}}{2}$, contradicting the axiom of equality, i.e. that $D_{b,c}=D_{b,c}$.

Therefore, by contradiction, $x=y$.

Therefore, $x=a$, proving that $P_{b,c}$ contains a node *a* such that $D_{a,b}=D_{a,c}$,

# Supplementary Figures

Supplementary Figure 1. A schematic illustrating the BLOBFISH algorithm, for $h=4$.

Supplementary Figure 2. Tissue-specific eQTL subnetworks for GTEx samples from (A) subcutaneous adipose, (B) skeletal muscle, (C) lung tissue, (D) skin, and (E) aorta.

Supplementary Figure 3. Tissue-specific gene regulatory subnetworks for GTEx samples from males aged 20-29 years in (A) lung tissue, (B) skin, and (C) aorta obtained using LIONESS+BLOBFISH.

Supplementary Figure 4. Tissue-specific gene regulatory subnetworks for GTEx samples from males aged 20-29 years in (A) subcutaneous adipose, (B) skeletal muscle, (C) lung tissue, (D) skin, and (E) aorta obtained using LIONESS+SP.

Supplementary Figure 5. Adipose-specific eQTL subnetwork for GTEx samples, including only genes associated with adipogenesis.

# Supplementary Tables

| Gene Symbol | Ensembl ID |
| --- | --- |
| MB | ENSG00000198125 |
| MYH2 | ENSG00000125414 |
| MYL2 | ENSG00000111245 |
| DES | ENSG00000175084 |
| TNNC1 | ENSG00000114854 |
| TNNC2 | ENSG00000101470 |
| ENO3 | ENSG00000108515 |
| MYL3 | ENSG00000160808 |
| TTN | ENSG00000155657 |
| TPM1 | ENSG00000140416 |
| TCAP | ENSG00000173991 |
| MYL1 | ENSG00000168530 |
| TPM3 | ENSG00000143549 |
| TPM4 | ENSG00000167460 |
| COX5B | ENSG00000135940 |
| MYL2 | ENSG00000111245 |
| COX5A | ENSG00000178741 |
| CKMT2 | ENSG00000131730 |
| TUBA1A | ENSG00000167552 |
| TUBA1B | ENSG00000123416 |
| TUBA4A | ENSG00000127824 |
| TUBA1C | ENSG00000167553 |
| TUBA3C | ENSG00000198033 |
| TUBA8 | ENSG00000183785 |
| TUBA3D | ENSG00000075886 |
| TUBA3E | ENSG00000152086 |
| TUBA4B | ENSG00000243910 |

Supplementary Table 1. Genes associated with skeletal muscle development.

| Gene Symbol | Ensembl ID |
| --- | --- |
| PPARG | ENSG00000132170 |
| FASN | ENSG00000169710 |
| SREBF1 | ENSG00000072310 |
| SCD | ENSG00000099194 |
| CEBPA | ENSG00000245848 |
| ADIPOQ | ENSG00000181092 |
| FABP4 | ENSG00000170323 |

Supplementary Table 2. Genes associated with adipogenesis.

| TF ID | TF gene | CHR | POS | Gene ID | Gene Name | Gene Type |
| --- | --- | --- | --- | --- | --- | --- |
| chr12_48386611_T_G_b38 | TUBA1B | 12 | 48386611 | ENSG00000293682 | ENSG00000293682 | lncRNA |
| chr12_48386611_T_G_b38 | TUBA1C | 12 | 48386611 | ENSG00000293682 | ENSG00000293682 | lncRNA |
| chr12_49672264_G_A_b38 | TUBA1B | 12 | 49672264 | ENSG00000161791 | FMNL3 | Protein coding |
| chr12_49672264_G_A_b38 | TUBA1A | 12 | 49672264 | ENSG00000161791 | FMNL3 | Protein coding |

Supplementary Table 15. eQTL network connecting genes associated with skeletal muscle development and/or adipogenesis exclusive to GTEx lung tissue samples.

| TF | TF gene | CHR | POS | Gene ID | Gene Name | Gene Type |
| --- | --- | --- | --- | --- | --- | --- |
| chr12_49537023_G_T_b38 | TUBA1A | 12 | 49537023 | ENSG00000287537 | ENSG00000287537 | lncRNA |
| chr12_49537023_G_T_b38 | TUBA1C | 12 | 49537023 | ENSG00000287537 | ENSG00000287537 | lncRNA |

Supplementary Table 17. eQTL network connecting genes associated with skeletal muscle development and/or adipogenesis exclusive to GTEx aorta samples.
